# Supplementary material for: Sociodemographic Factors Influencing the Use of eHealth in People with Chronic Diseases
Source: Int J Environ Res Public Health. 2019 Feb 21;16(4):645. doi: 10.3390/ijerph16040645 (PMC6406337; doi:10.3390/ijerph16040645)
Supplement: Supplementary file 1 [file ijerph-16-00645-s001.zip › Suppl.Mat. Quality assessment MMAT.docx]

| **Qualitative** | **1.1.** | **1.2.** | **1.3.** | **1.4.** | **1.5.** |
| --- | --- | --- | --- | --- | --- |
| Smith et al. | yes | no | yes | Can’t tell | yes |
| **Quantitative randomised controlled trials** | **2.1.** | **2.2.** | **2.3.** | **2.4.** | **2.5.** |
| Hanberger et al. | yes | yes | yes | Can’t tell | no |
| Rixon et al. | yes | yes | no | Can’t tell | yes |
| **Quantitative nonrandomized** | **3.1.** | **3.2.** | **3.3.** | **3.4.** | **3.5.** |
| Drewes et al. | yes | no | yes | no | yes |
| LaMonica | no | yes | yes | yes | yes |
| Nelson et al. | yes | yes | yes | yes | yes |
| Rho et al. | yes | yes | yes | Can’t tell | yes |
| Song et al. | yes | No | yes | yes | yes |
| Pollom et al | yes | no | no | no | yes |
| Duplaga et al. | Can’t tell | yes | yes | yes | yes |
| Edwards et al | yes | yes | yes | yes | yes |
| Goyal et al. | no | no | yes | Can’t tell | yes |
| **Quantitative descriptive** | **4.1.** | **4.2.** | **4.3.** | **4.4.** | **4.5.** |
| Anglada-Martinez et al. | no | yes | yes | Can’t tell | yes |
| Drewes et al. | yes | no | yes | no | yes |
| Han et al. | yes | yes | yes | yes | yes |
| Hofstede et al. | yes | yes | yes | yes | yes |
| Kamis et al. | yes | yes | yes | no | yes |
| Saieda et al. | yes | no | Can’t tell | No | yes |
| Samiei et al. | yes | yes | yes | yes | yes |
| Sarkar et al. | yes | yes | yes | no | yes |
| Terschüren et al. | Yes | yes | yes | Can’t tell | yes |
| Whittemore et al. | yes | yes | yes | no | yes |
| **Mixed methods** | **5.1.** | **5.2.** | **5.3.** | **5.4** | **5.5** |
| Jacobs et al. | yes | yes | yes | yes | yes |

**Supplemental Material : quality assessment table according to MMAT**
