# Supplementary material for: Sociodemographic Factors Influencing the Use of eHealth in People with Chronic Diseases
Source: Int J Environ Res Public Health. 2019 Feb 21;16(4):645. doi: 10.3390/ijerph16040645 (PMC6406337; doi:10.3390/ijerph16040645)
Supplement: Supplementary file 1 [file ijerph-16-00645-s001.zip › Supplemental Material-combin.search.terms.docx]

|  | Groups entered | filters used |
| --- | --- | --- |
| PubMed | Group 1 AND 2 AND 3 AND 4 | group 2: title  excluding reviews  last 10 years |
| CINAHL | Group 1 AND 2 AND 3 AND 4 | group 2: title  excluding reviews  last 10 years |
| ScienceDirect | Group 1 AND 2 and several terms of group 3 and 4  (just 8 Boolean operators allowed)  terms:  adoption OR implementation OR intervention AND factors OR barriers OR characteristics OR facilitators OR predictors OR disparities  title/abstract: chronic diseases OR cardiovascular disease OR diabetes OR cancer OR chronic respiratory disease OR long-term disease OR heart disease OR dementia OR obesity  title: eHealth OR electronic devices OR “Web 2.0” OR net health OR “digital health technology” OR telemonitoring OR mHealth OR telehealth OR “health technology” | research articles  last 10 years  group 2: title/abstract/keywords  group 1: title |
| ACM Digital Library | Group 1 AND 2 and several terms of group 3 and 4 | excluding reviews  last 10 years  group 1: abstract  group 2: title |

**Appendix A : Combination of search terms according to the database used**
