# Supplementary material for: Sociodemographic Factors Influencing the Use of eHealth in People with Chronic Diseases
Source: Int J Environ Res Public Health. 2019 Feb 21;16(4):645. doi: 10.3390/ijerph16040645 (PMC6406337; doi:10.3390/ijerph16040645)
Supplement: Supplementary file 1 [file ijerph-16-00645-s001.zip › Suppl. file - Sociodemographic factors influencing the use of eHealth and suggested interventions..docx]

**Table 2.** Overview of included articles and data extracted .Sociodemographic factors influencing the use of eHealth and suggested interventions.

| **Author and Publication Year** | **Country** | **Study Design** | **Aim of the Study** | **Technology Used** | **Disease for Which eHealth is Used** | **Factors Influencing Use of eHealth** | **Factors Not Influencing Use of eHealth** | **Suggested Interventions to Improve the Use of eHealth** |
| --- | --- | --- | --- | --- | --- | --- | --- | --- |
| Anglada-Martínez et al. 2016 | Spain | Quantitative descriptive | Use | Smartphone application | Heart failure, hypertension, dyslipidemia | - Age | - Gender | - eHealth education - Involving caregivers |
| Drewes et al. 2016 | Germany | Quantitative descriptive | Acceptance | eHealth technologies | Breast cancer | - Age - Multiple-person household | - Education - Vocational status | - Tailor the implementation of eHealth to patients’ individual needs |
| Duplaga et al. 2015 | Poland | Quantitative non-  randomized | Acceptance | eHealth technologies | Chronic diseases | - Age - Place of residence | - Gender | - Appropriate preparation of target audiences |
| Edwards et al. 2014 | England | Quantitative descriptive | Interest | eHealth technologies | Cardiovascular disease or depressions | - Age - Place of residence | - Gender - Ethnicity | - |
| Goyal et al. 2016 | Canada | Quantitative non-  randomized | Willingness | Smartphone application | Chronic diseases | - Age - Gender | - | - Offering customization by presenting users with challenges tailored to their profile |
| Han et al. 2010 | Australia | Quantitative descriptive | Effectiveness | eHealth technologies | Chronic diseases | - Place of residence - Socio-economic status - Income | - | - Formulate eHealth strategies to respond to the specific target population |
| Hanberger et al. 2013 | Sweden | Randomized Controlled Trial (RCT) | Use | Web portal | Children with type 1 diabetes | - Gender | - | - |
| Hofstede et al. 2014 | Netherlands | Quantitative descriptive | Use | eHealth applications | Asthma or COPD^1^ | - Age - Education | - | - Give older patients and persons with lower educational level extra support |
| Jacobs et al. 2018 | United States | Mixed methods | Access | Mobile system | Breast cancer | - Place of residence | - | - Make survey completion task easy - Not overwhelming participants with too much information - Make use of their desire to help future patients. |
| Kamis et al. 2015 | Bolivia | Quantitative descriptive | Access | mHealth | Chronic diseases | - Age - Gender - Education | - | - Receive a mobile phone call free of charge |
| LaMonica et al. 201 | Australia | Quantitative non-  randomized | Use | eHealth technologies | SCI^2^, MCI^3^,  dementia | - Age - Education | - Vocational status | - Support novice users - Promote the use and uptake of social media - Use texts: easy and cheap - Use mobile-friendly websites - Use eHealth offline - Adapt eHealth to the ability of the patient |
| Nelson et al. 2015 | United States | Quantitative non-  randomized | Engagement | mHealth | Type 2 diabetes | - Age - Ethnicity | - Gender - Income - Health literacy | - Tailor content to cultural attitudes/beliefs - Involve family members - Customized mHealth to different user groups - Using participatory design methods with racial/ethnic minorities |
| Rho et al. 2017 | South Korea | Quantitative non-  randomized | Use | eHealth technologies | Diabetes | - Place of residence | - Age - Gender | - |
| Rixon et al. 2013 | England | RCT | Use | Telehealth | COPD, diabetes,  heart failure | - Education | - Age - Gender | - |
| Saied et al. 2014 | United States | Quantitative descriptive | Use | Internet-based devices | Cancer | - Age - Place of residence | - Gender | - Use more communication methods, not just eHealth |
| Samiei et al. 2016 | Malaysia | Quantitative descriptive | Interest | Internet-based program | Type 2 diabetes | - Age - Education - Income | - Gender - Ethnicity | - Increase Internet access - Subsidizing the cost of computer devices |
| Sarkar et al. 2008 | United States | Quantitative descriptive | Interest | eHealth technologies | Diabetes | - | - Ethnicity - Age - Education - Health literacy | - Offering different support services to meet the needs of their diverse patient populations |
| Smith et al.  2015 | India | Qualitative | Use | mHealth | Cardiovascular disease | - Age | - | - mHealth as a complement, rather than replace existing methods of healthcare delivery - Using text messages to provide lifestyle advice and health awareness - Calling is preferred in low literacy patients - Involving family - Support from health care workers |
| Song et al. 2017 | United States | Quantitative non-  randomized | Use | eHealth technologies | Prostate cancer | - | - eHealth literacy of partners - Age - Education - Ethnicity - Income | - Also provide educational information and materials that are not electronically based - Encourage patients and their partners with low eHealth literacy - Involve family members with high eHealth literacy - Provide tablet when waiting for an appointment or during a treatment session |
| Terschüren et al. 2012 | Germany | Quantitative descriptive | Awareness | Telemedical devices | Diabetes, heart disease | - Education - Gender - Age | - Place of residence | - Immediate trusted translation of data into “alert” or “all-clear” is needed - Awareness of eHealth by elderly through television and magazines - Combination of telecare and qualified practice assistants |
| Whittemore et al.  2013 | United States | Quantitative descriptive | Willingness | Internet-based programs | Type 1 diabetes | - Income - Ethnicity - Gender | - Age | - Race disparities: creative recruitment approaches are needed - Use social media to reach diverse youth - Involving targeted users in the design and development of programs - Keep text to a minimum - Movie material with people presenting the same race - Use multiple platforms, such as the Internet and smartphones - Involve parents; obtaining information on parents regarding a proposed eHealth intervention |
| Pollom et al.  2015 | United States | Quantitative non-  randomized | Feasibility | Tablet | Head or neck cancer | - Age | - Marital status - Gender | - More assistance for elderly, allow more time for elderly - Built-in tutorials and added on-site assistance |

1 COPD: chronic obstructive pulmonary disease; 2 SCI: subjective cognitive impairment; 3 MCI: mild cognitive impairment.
